# Editorial

**Published:** 1899-02

**Authors:** 


### Editorial.

The Tri-State Association of Mississippi, Arkansas and Tennessee takes up the cudgel in behalf of Parke, Davis & Co. and the *Medical Age* of Detroit, as follows:

"Whereas, The medical laws of the various States have been so perverted by political influences as to give legislative sanction to grotesque, ignorant and dangerous sects of pretenders and charlatans; and

Whereas, The privileges granted to one of the most outrageous aberrations, namely, the so-called osteopathy, constitute a disgrace to the States in which the "osteopathists" are legally intrenched; and

Whereas, A certain William Smith, osteopathist, having been roundly denounced, together with his sect, by Parke, Davis & Co. and the *Medical Age*, now brings suit against both for $25,000 damages; therefore,

Be it declared the sentiment of the Tri-State Medical Association, of Mississippi, Arkansas and Tennessee, That Parke, Davis & Co. and the *Medical Age* are entitled to the sympathy of its members and of all medical practitioners; that we wish and expect them to enjoy a complete triumph in repelling this legal assault; and that wheresoever a powerful house takes a bold stand in opposition to quackery it promotes the interests of legitimate and honorable medicine and the welfare of humanity."

To those of our readers who are not familiar with this new comer from Edom, we would explain that osteopathy is a species of medical heterodoxy erected upon the assumption that all disease is dependent upon some skeletal derangement, the correction of which will cause the disease to disappear. We can not attempt to explain anything further concerning this singular system. It is gaining ground in the West very naturally, as this section of the country has always been noted for the peculiarity of its products from John Brown to Jerry Simpson. It has its official organs, one of which is backed by a paralytic ex-Congressman or justice of the peace, the former recipient of some public office of sufficient gravity to warrant the prefix "Hon." It numbers among its adherents not a few ladies, whose features as depicted in their journals modify the acerbity of the text. As a matter of course, osteopathy has received clerical sanction, for what system of charlatanry is complete without the endorsement of some pilot of the straight and narrow way?

It is a curious fact that to no class does quackery appeal so powerfully as to the clergy, a circumstance suggestive of either the theurgic origin of most quackeries or the ancient connection between priestcraft and medicine.

The osteopaths claim a curriculum of study equal to that of the regular medical schools, though the standing of their graduates before the State examining boards does not bring out the truth of this assertion with startling distinctness.

In all these aberrations, it is not hard to prognosticate their fate. They become, sooner or later, additions to the pile of rubbish accumulated during the ages of worn-out theories and wrecked and broken systems growing out of the folly and credulity of man and owing their destruction to the disintegrating effect of scientific truth when applied to shams.

Looking broadly at this and kindred concerns, the medical body is no more harmed by these barnacles that cling to it than is the sperm whale by the marine life which flourishes on its broad back. Its nutrition is not jeopardized; its strength and the use of it is not crippled. The parasites are mere saprophytes and are innocuous.

The Philistine intelligence demands something more than unreasonable *ipse dixits*. There will always be need of good surgeons and doctors and specialists in their several branches who practice their art openly and sensibly without any slobbering nonsense and abracadabra rot. To these the intelligent class of the community will turn. The others will naturally gravitate toward that system adapted to their intellectual level, whether that be found in Christian Science, Osteopathy or Voodooism.

Give every system and sect and creed a hearing so long as it is not harmful or criminal, and then submit each to the extraordinary question of an examination before a medical board. If they pass this, then let them alone, for if a "beast be lord of beasts, his crib shall stand at the king's mess." Let the medical examining board be the supreme test of fitness. It is the surest factor for repressing ignorance and unfitness and does more good than all the anathema maranathas hurled at the irregulars.

"Let them alone and they'll come home
And leave their tails behind them"—

safe in the pathological collection of the examiners.

---

### "TAKE ONE."

We learn that our esteemed contemporary, the *Atlanta Medical and Surgical Journal*, has been appointed the official organ of the Atlanta Society of Medicine. This was done in consideration of our e. c.'s generous offer of publishing the proceedings of that respectable but somewhat decrepit body free, to include a year's subscription to every member. Does the indestructibly indexed clinical thermometer go along with this prize package, or must one send five two-cent stamps to procure it, postage paid?

It is a grief to see our dignified old friend putting itself on the level of the *Medical Bluff* in becoming a gift publication. It displays, however, a beautiful spirit of philanthropy to send abroad the glad tidings of help and succor for afflicting humanity without money and without price. We find it expensive to carry on a medical journal and have not yet reached that eminence, apparently attained by our e. c., which would place us above the sordid consideration of dollars and cents. We are constrained to pursue the fugacious if almighty dollar, in the blind prejudice that if our readers want onr journal they will be willing to pay for it.

---

Dr. W. C. Jarnigan has been elected a member of the Board of Health of Atlanta to succeed Dr. J. C. Avary, resigned.

---

The death of Dr. G. H. Robie, of Maryland, and Dr. J. H. Ethridge, of Chicago, is announced.

---

Mr. C. B. Kirkland has severed his connection with Parke, Davis & Co., and is now with the advertising department of the J. C. Ayer Co., Lowell, Mass.

